# The dual impact of physical exercise on university students’ mental health: the chain mediating effects of mindfulness and psychological resilience

**DOI:** 10.3389/fpsyg.2025.1545370

**Published:** 2025-04-14

**Authors:** Jian Guo

**Affiliations:** Department of Physical Education, Liaoning University of Technology, Jinzhou, China

**Keywords:** physical exercise, mindfulness, psychological resilience, mental health, university students, the dual-factor model of mental health, the conservation of resources theory

## Abstract

**Introduction:**

Mental health issues among university students are increasingly prominent, and effective interventions are urgently needed. Physical exercise has shown potential in improving mental health, but the underlying mechanisms remain unclear. Based on the Conservation of Resources (COR) theory and the Dual - Factor Model of Mental Health, this study aims to explore the dual impact of physical exercise on university students’ mental health and the chain - mediating effects of mindfulness and psychological resilience.

**Methods:**

A cross-sectional survey was carried out on 720 students from Chinese universities. Validated instruments were used to measure physical exercise, mindfulness, resilience, and mental health outcomes. SPSS 27.0 and Mplus 8.3 were employed for data analysis, including descriptive statistics, correlation analysis, confirmatory factor analysis, and mediation effect testing.

**Results:**

The findings show that physical exercise is a proactive resource investment behavior. It significantly enhances students’ mindfulness and resilience. These psychological resources promote positive mental health indicators such as life satisfaction and positive affect, and at the same time, reduce negative factors like psychological distress. The chain mediation analysis indicates that mindfulness and resilience act as interconnected resources, which is in line with the “gain spiral” and “resource caravan” effects in the COR theory.

**Discussion:**

This study provides novel insights by demonstrating how mindfulness and resilience sequentially amplify the psychological benefits of physical exercise. It offers a more detailed understanding of the mechanisms behind the improvement of university students’ mental health. The results have significant theoretical and practical implications, advocating for the integration of exercise, mindfulness, and resilience-building strategies in mental health interventions for university populations.

## 1 Introduction

University years represent a pivotal developmental stage, transitioning individuals from late adolescence to early adulthood (Cuijpers et al., 2019). During this period, students’ mental health significantly influences not only academic performance but also long-term social adaptability and career success (Liu et al., 2019). A World Health Organization survey reported that 20.3% of university students experienced DSM-IV/CIDI disorders^1^ in the past year, with anxiety and mood disorders being most prevalent (Auerbach et al., 2016). Recent findings reveal that the COVID-19 pandemic exacerbated these issues, raising the prevalence of depression and anxiety among students to 39 and 36%, respectively (Li Y. et al., 2021). These trends highlight the urgent need for effective mental health interventions tailored for university students.

Physical exercise, as a non-pharmacological intervention, has gained increasing attention for its multidimensional benefits. Research demonstrates its effectiveness in improving physiological health and mental states, including reducing anxiety, stress, and depression through mechanisms such as endorphin release and neurotransmitter regulation (Mikkelsen et al., 2017). Furthermore, physical activity enhances life satisfaction and well-being, which is especially vital for students navigating this transitional life phase (Herbert, 2022). However, while numerous studies have explored the physiological and psychological pathways linking exercise to mental health (Doré et al., 2020; Mahindru et al., 2023), the interaction remains complex, requiring further exploration of multidimensional mechanisms (Xu, 2020).

To further elucidate the complex mechanisms through which physical exercise influences mental health, the Conservation of Resources (COR) theory provides an ideal theoretical framework. Proposed by Hobfoll (1989), this theory posits that individuals strive to acquire, retain, and protect valuable resources to cope with stress. These resources encompass various domains of life, including personal resources (e.g., interpersonal relationships), psychological resources (e.g., self-efficacy), and material resources (e.g., financial assets) (Hobfoll, 1989; Siddiquei et al., 2025). The theory emphasizes that when individuals successfully obtain and conserve resources, these resources generate a gain effect, forming a “gain spiral” (Hobfoll, 2001). This positive resource feedback enhances individuals’ coping abilities, mitigates stress, and improves mental health. Additionally, Hobfoll (2011) further introduced the concept of “resource caravans,” suggesting that different types of resources are interconnected, interdependent, and function collectively.

From the perspective of the COR theory, the stressors faced by university students—such as academic competition, social adaptation, and the impact of the COVID-19 pandemic—essentially constitute a persistent threat to personal resources. Such threats may lead to resource depletion, which, in turn, triggers mental health issues. However, physical exercise serves as an effective resource investment behavior, not only improving physiological well-being but also facilitating the acquisition and accumulation of key psychological resources. This process buffers the negative effects of stress and enhancing overall well-being (Kettunen et al., 2015). The COR theory has been widely used to explain how individuals’ mental health is influenced by resource loss or gain when facing stress and challenges (Kettunen et al., 2015; Egozi Farkash et al., 2022; Mubarak et al., 2022), yet few studies have applied it to explore the mechanisms by which exercise affects university students’ mental health.

Additionally, according to COR theory, mindfulness and psychological resilience are two critical psychological resources that can enhance an individual’s ability to cope with environmental stressors and mitigate the detrimental effects of psychological distress (Hobfoll, 2011; Mubarak et al., 2022). Mindfulness helps individuals effectively manage negative emotions and stressors, reducing resource depletion and enhancing the preservation and regulation of psychological resources (Mubarak et al., 2022). Psychological resilience, as a core resource for coping with adversity and stress, enables individuals to effectively mobilize internal and external resources to cope with various life pressures and challenges (Zhu et al., 2019). Furthermore, studies have shown that mindfulness-based interventions can enhance resilience and help students manage the various stressors of modern society (Bajaj and Pande, 2016; Arpaci and Gundogan, 2020). Therefore, mindfulness and psychological resilience may exhibit a sequential relationship.

Mindfulness and psychological resilience have emerged as critical research variables in recent years, demonstrating significant effects on promoting mental health (Hu et al., 2015; Tran et al., 2022). However, empirical studies on how physical exercise affects university students’ mental health through these psychological resources remain limited. This study employs the COR theory to investigate the mediating effects of mindfulness and psychological resilience in the relationship between physical exercise and mental health among university students, aiming to advance our understanding of the mechanistic pathways through which physical exercise influences mental health.

### 1.1 Physical exercise in relation to mindfulness and psychological resilience

Within the framework of the COR theory, physical exercise is not only a proactive resource investment behavior but also an effective means of accumulating psychological resources. Mindfulness, defined as “the awareness that emerges through paying attention on purpose, in the present moment, and nonjudgmentally to the unfolding of experience moment by moment” (Kabat-Zinn, 2003), enables individuals to manage distressing thoughts and emotions effectively (Brown et al., 2007). Modern psychology posits that “present-focused attention” and “non-judgmental attitude” toward the object of attention are two essential psychological factors of mindfulness (Peng and Ju, 2013). During physical exercise, an individual’s awareness of internal bodily sensations and attention to the exercise context, along with an objective self-assessment, acceptance of others, and a composed response to competition outcomes, exhibit high similarity to the mindfulness factors of “present-focused attention” and “non-judgmental attitude.” This similarity suggests a strong theoretical alignment between the psychological structure of physical exercise and that of mindfulness. Empirical research also supports this perspective. Studies by Xu (2021) and Caldwell et al. (2010) have found that active participation in physical exercise significantly enhances mindfulness levels among university students.

Furthermore, psychological resilience is defined as “the process of adapting well in the face of adversity, trauma, tragedy, threats or even significant sources of stress” (Southwick et al., 2014). The COR theory emphasizes that when individuals face stress, they must mobilize and protect their resources (Hobfoll, 1989). Psychological resilience, as a key personal resource, enhances an individual’s ability to effectively utilize internal and external resources, enabling them to adapt more efficiently to adversity (Mubarak et al., 2022; Siddiquei et al., 2025). The unique characteristics of physical exercise environments—such as enjoyment, openness, and competitiveness—create favorable conditions for fostering psychological resilience in university students. Physical activities involve rapid and intense shifts in space, time, and exercise intensity, requiring students to adapt quickly to dynamic, unpredictable changes, thereby training their responsiveness and information-processing abilities (Li, 2017). Such training enhances students’ ability to mobilize internal and external resources, thereby strengthening their resilience to stress and adversity. Research further indicates that increased exercise volume (Román-Mata et al., 2020) and intensity (Dunston et al., 2020) significantly boost psychological resilience levels among university students. Moreover, neuroscience research confirms that levels of brain-derived neurotrophic factor significantly increase during physical exercise, which protects neurons in the striatum and hippocampus under stress, thus positively influencing psychological resilience (Russell et al., 2014). These findings suggest that physical exercise not only directly facilitates the development of psychological resilience but also constructs a solid psychological defense for individuals through mechanisms of resource accumulation and regeneration.

### 1.2 Mindfulness and psychological resilience in relation to mental health

Within the framework of COR theory, mindfulness, as a key personal psychological resource, helps individuals identify and regulate emotions in stressful situations, thereby reducing resource depletion and enhancing resource preservation mechanisms (Mubarak et al., 2022). Evidence shows that high levels of mindfulness help university students reduce loneliness and cope with stress, anxiety, and depression (Dvořáková et al., 2017; Jin et al., 2020; González-Martín et al., 2023). For instance, randomized controlled trials demonstrate that mindfulness training significantly enhances life satisfaction and reduces psychological distress (Dvořáková et al., 2017). Additionally, the systematic review and meta-analysis conducted by González-Martín et al. (2023) further validated the significant effects of mindfulness interventions in improving mental health among university students. Therefore, cultivating mindfulness helps to prevent psychological resource depletion, enabling individuals to better maintain mental well-being when facing stress.

Furthermore, within the framework of COR theory, psychological resilience is also regarded as a key personal psychological resource, which plays a crucial role in protecting and regenerating resources when individuals face external resource threats (Egozi Farkash et al., 2022; Mubarak et al., 2022; Zhu et al., 2019). A growing body of empirical research suggests that university students with higher levels of psychological resilience tend to exhibit better mental health, greater overall well-being, and significantly lower levels of depression and anxiety when coping with academic stress and life challenges (Davydov et al., 2010; Hu et al., 2015; Antonini Philippe et al., 2021). These findings indicate that in stressful contexts, psychological resilience can effectively protect and enhance individuals’ psychological resources, ultimately exerting a positive impact on mental health.

### 1.3 Mindfulness in relation to psychological resilience

As a positive psychological resource, mindfulness not only helps individuals regulate emotions when facing stress but also supports resource conservation, thus promoting the cultivation of psychological resilience. Research indicates that mindfulness significantly enhances psychological resilience, particularly in helping university students manage academic and life pressures. For instance, Bajaj and Pande (2016) found that undergraduate students with higher mindfulness levels exhibited greater resilience. Similarly, Arpaci and Gundogan (2020) demonstrated that psychological resilience mediates the relationship between mindfulness and nomophobia, suggesting that mindfulness-based interventions could bolster resilience to help students cope with various stressors in modern society. Moreover, mindfulness reduces maladaptive coping mechanisms, such as avoidance and rumination, which prevents the onset of depressive thinking and enhancing resilience (Thompson et al., 2011). In educational contexts, Zenner et al. (2014) systematically reviewed school-based mindfulness interventions, finding significant improvements in cognitive performance and stress resilience among youth. These findings support the potential application of mindfulness interventions in university settings. Collectively, these findings highlight a sequential relationship between mindfulness and psychological resilience, emphasizing its broad applicability in improving mental health outcomes in university students.

### 1.4 The dual-factor model of mental health

This study employs the Dual-Factor Model of Mental Health (DFM) to comprehensively examine the role of mindfulness and psychological resilience in the mechanisms through which physical exercise influences university students’ mental health. Unlike traditional models, which view happiness and psychopathology as opposite ends of a single continuum (Suldo and Shaffer, 2008), the DFM, developed within the framework of positive psychology, considers distress and well-being as distinct yet interrelated constructs (Keyes, 2002; Suldo and Shaffer, 2008; Antaramian et al., 2010). This model underscores that mental health encompasses not only the absence of psychopathological symptoms but also the presence of positive psychological states, such as subjective well-being and life satisfaction (Keyes, 2005; Suldo and Shaffer, 2008; Westerhof and Keyes, 2010). This dual perspective is particularly pertinent for university students, whose mental health often reflects a coexistence of distress and well-being. For example, they may maintain optimism about the future while navigating academic pressures or experience personal growth and satisfaction despite life challenges. Thus, evaluating university students’ mental health requires a holistic approach that considers both positive and negative factors to provide a more comprehensive understanding (Greenspoon and Saklofske, 2001; Hu et al., 2015).

Although the DFM has been widely applied in mental health research (Antaramian, 2015; Zhang et al., 2021), current studies on the effects of physical exercise, mindfulness, and resilience on mental health often focus on a single dimension (Li X. N. et al., 2021; Wang et al., 2022), overlooking the comprehensive impact of both positive and negative dimensions. Given the complex relationship between physical exercise and university students’ mental health, this study integrates the COR theory and the DFM to comprehensively explore the mechanisms through which physical exercise influences mental health through mindfulness and psychological resilience, and further reveals their chain mediation effects.

**Figure 1:**
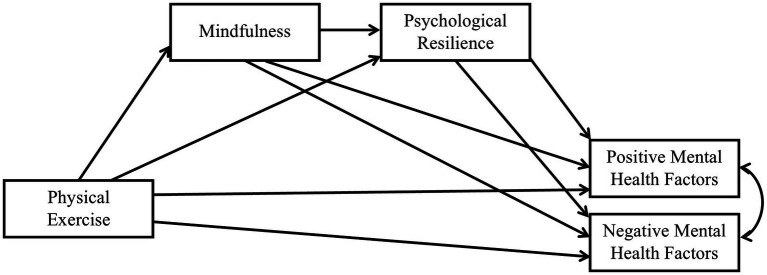
Conceptual framework.

Based on the above analysis, we developed a conceptual model (as shown in Figure 1).

## 2 Materials and methods

### 2.1 Participants

The study randomly selected five universities in Liaoning Province, China, as the sampling source between June 15 and June 23, 2024. Two classes from each grade were randomly selected at each university, resulting in a total of 40 classes as the study sample. Data were collected using the online survey platform “Wenjuanxing,” where electronic questionnaires were distributed to participants. A total of 834 questionnaires were received. After excluding invalid responses—such as patterned answers, duplicate submissions from the same IP address, and responses completed in less than 5 min—720 valid questionnaires were retained, yielding an effective response rate of 86.33%.

Among the valid respondents, 336 participants (46.67%) were male, and 384 (53.33%) were female. The grade distribution included 186 freshmen (25.83%), 197 sophomores (27.36%), 181 juniors (25.14%), and 156 seniors (21.67%). Regarding birthplace, 283 participants (39.31%) were from urban areas, while 437 (60.69%) were from rural areas. In terms of only-child status, 292 participants (40.56%) were only children, while 428 (59.44%) had siblings. Participants ranged in age from 17 to 24 years, with 43.19% aged 17–19, 54.86% aged 20–22, and 1.94% aged 23–24. The average age was 19.77 years (SD = 1.31), and the average body mass index (BMI) was 23.30 (SD = 6.76). For a complete overview of demographic characteristics, see Table 1.

**Table 1:** Demographic characteristics of participants.

Demographic variables	Category	*N* (%)
Gender	Male	336 (46.67)
	Female	384 (53.33)
Only-child status	Yes	292 (40.56)
	No	428 (59.44)
Age	17–19	311 (43.19)
	20–22	395 (54.86)
	23–24	14 (1.94)
Birthplace	Urban	283 (39.31)
	Rural	437 (60.69)
Grade	Freshman	186 (25.83)
	Sophomore	197 (27.36)
	Junior	181 (25.14)
	Senior	156 (21.67)

### 2.2 Procedure

This study employed a cross-sectional survey design, with data collected via the online platform Wenjuanxing. Members of the research team explained the study’s purpose and requirements to class advisors, who subsequently assisted in distributing the survey link to students. Participants completed the survey on-site using their mobile phones. The entire survey process was supervised by the research team to ensure the authenticity of the responses and the accuracy of the data. After the survey, data were exported to SPSS for cleaning and analysis, and invalid responses were excluded to ensure data validity and reliability. Before participation, all students were informed about the purpose and content of the study and participated voluntarily after signing an informed consent form in accordance with the Declaration of Helsinki. Participant anonymity was ensured throughout the data collection process, and the study strictly adhered to ethical guidelines for psychological research.

### 2.3 Measures

#### 2.3.1 Physical exercise scale for university students

This study employed the *Chinese College Students’ Physical Exercise Scale* revised by Wu (2016). The instrument has been widely applied among Chinese university students and has demonstrated high reliability and validity within this population (Jiang et al., 2018; Li X. N. et al., 2021). The scale consists of eight items, divided into two dimensions: exercise adherence (four items) and exercise commitment (four items). Responses are rated on a 5-point Likert scale, where 1 represents “strongly disagree” and 5 represents “strongly agree.”

Confirmatory factor analysis (CFA) revealed that one item’s factor loading in the exercise commitment dimension was below 0.7, leading to its removal. The adjusted CFA results indicated good model fit: *x*^2^/*df* = 6.38, CFI = 0.98, TLI = 0.97, RMSEA = 0.09, SRMR = 0.02. CFI and TLI values greater than 0.95 indicate good model fit, and the RMSEA value, close to the threshold of 0.08, is within the acceptable range (Jackson et al., 2009). In this study, the Cronbach’s *α* for the overall scale was 0.92, with values of 0.88 and 0.89 for the exercise adherence and exercise commitment subscales, respectively.

#### 2.3.2 General health questionnaire-12 (GHQ-12)

This study employed the GHQ-12 to assess the negative aspects of university students’ mental health. The GHQ-12 is widely used to screen for psychological distress and common mental health issues, as well as to identify potential cases of mental disorders (Makowska and Merecz, 2000; Romppel et al., 2013). The version utilized in this study is the Chinese adaptation revised by Zhang et al. (2008), which has been extensively applied to evaluate the mental health of Chinese university students (Shu et al., 2021; Tang et al., 2021).

The GHQ-12 comprises 12 items, requiring participants to reflect on their experiences over the past 4 weeks. Each item is rated on a 4-point Likert scale ranging from 1 (“never”) to 4 (“almost always”). Six items are positively worded and reverse-scored, while the other six are negatively worded and scored normally. The total GHQ-12 score ranges from 12 to 48, with higher scores indicating greater psychological distress. In this study, the Cronbach’s *α* for the GHQ-12 was 0.87, demonstrating good internal consistency.

#### 2.3.3 Subjective well-being

Subjective well-being, a positive indicator of mental health, consists of both cognitive and emotional components (Antaramian, 2015). In this study, the cognitive component of subjective well-being was measured using the *Satisfaction with Life Scale* (SWLS) (Diener et al., 1985). The SWLS is a widely utilized instrument for evaluating an individual’s overall life satisfaction and includes of five items, each rated on a 7-point Likert scale ranging from 1 (“strongly disagree”) to 7 (“strongly agree”). The total SWLS score ranges from 5 to 35, with higher scores indicating greater life satisfaction. Studies by Jiang et al. (2023) and Bieda et al. (2019) have demonstrated the cultural validity and applicability of the SWLS among Chinese university students. In this study, the Cronbach’s α for the SWLS was 0.88, indicating good internal consistency.

The emotional component of subjective well-being was assessed using the *Positive and Negative Affect Schedule* (PANAS). The PANAS, developed by Watson et al. (1988), was adapted for Chinese university students by Qiu et al. (2008). The revised scale comprises of 18 emotion-related words, with nine representing positive affect (PA) and nine representing negative affect (NA). Participants rated their emotional experiences over the past week on a 5-point Likert scale, ranging from 1 (“not at all”) to 5 (“extremely”). Higher scores reflect more frequent emotional experiences. In this study, the Cronbach’s *α* for the PA and NA scales was 0.96 and 0.93, respectively, indicating excellent internal consistency.

Following previous studies (Antaramian et al., 2010; Thrash et al., 2010; Ye et al., 2019), the composite score for subjective well-being in this study was calculated by adding the standardized SWLS score to the standardized PA score and subtracting the standardized NA score. This approach enables a comprehensive evaluation of both the cognitive and emotional components of subjective well-being, providing a more holistic assessment of university students’ mental health status.

#### 2.3.4 Mindful attention awareness scale (MAAS)

This study employed the MAAS to evaluate the mindfulness levels of university students. The MAAS was developed by Brown and Ryan to measure an individual’s awareness of the present moment during daily activities, reflecting their level of mindfulness (Brown and Ryan, 2003). The MAAS is a unidimensional scale encompassing various aspects of mindfulness, including cognitive, emotional, and physiological experiences in daily life.

The Chinese version of the MAAS used in this study was revised by Chen et al. (2012) and has been extensively applied in mindfulness research within the Chinese cultural context, demonstrating high reliability and validity. The scale comprises 15 items, each rated on a 6-point Likert scale ranging from “always” (1 point) to “never” (6 points), capturing the degree of awareness individuals have of their daily experiences. The total mindfulness score is calculated by summing the scores of all items, with higher scores reflecting greater mindfulness, indicated by more frequent awareness and attention to present experiences. In this study, the Cronbach’s *α* for the MAAS was 0.91, indicating excellent internal consistency.

#### 2.3.5 Brief resilience scale (BRS)

This study utilized the BRS to evaluate the psychological resilience levels of university students. Developed by Smith et al. (2008), the BRS is designed to directly measure resilience by focusing on individuals’ ability to recover from stress and adversity rather than emphasizing coping strategies or available resources. Compared to other resilience scales, the BRS highlights the core of resilience—the capacity to bounce back from stress and setbacks. Previous research shows strong correlations between the BRS and other widely used resilience scales, such as the *Connor-Davidson Resilience Scale* (CD-RISC). Additionally, the BRS has demonstrated high reliability and validity in various studies (Smith et al., 2008; Chen et al., 2020).

In this study, the Chinese version of the BRS adapted by Chen et al. (2020) was employed. This version has been extensively used among Chinese university students and has demonstrated good reliability and validity (Chen et al., 2020; Fung, 2020). The BRS comprises six items, each rated on a 5-point Likert scale ranging from 1 (“strongly disagree”) to 5 (“strongly agree”). Three items are positively scored, while the other three are reverse-scored. The total resilience score is calculated by summing all six items. Higher scores reflect greater resilience, meaning a stronger ability to recover from stress and adversity. In this study, the Cronbach’s *α* for the BRS was 0.72, reflecting acceptable internal consistency.

### 2.4 Data analysis

Data analysis was conducted using SPSS 27.0 and Mplus 8.3. SPSS 27.0 was utilized for descriptive statistics, correlation analysis, and normalization of the data through z-scores. Mplus 8.3 was employed to perform CFA to examine model fit and mediation effects. The bias-corrected bootstrap method was adopted to test mediation effects. Compared to the traditional Sobel test, the bias-corrected bootstrap method provides higher statistical power when estimating indirect effects (Taylor et al., 2007; Fang and Zhang, 2012). In this study, the bootstrap method was based on 5,000 resamples, and a 95% confidence interval (CI) was used for significance testing. Mediation effects were considered significant if the CI did not include zero. The significance level was set at 0.05.

## 3 Results

### 3.1 Common method bias test and control

This study relied on self-reported data, which may introduce common method bias (CMB). To mitigate the potential impact of CMB on the results, several control measures were implemented during the research process, following the guidelines of Zhou and Long (2004). These measures included ensuring participant anonymity, clarifying that the data would be used exclusively for scientific research, and reverse-scoring certain items.

To further enhance the rigor of the study, Harman’s single-factor test was performed prior to data analysis to assess common method bias (Podsakoff et al., 2003). Specifically, an unrotated principal component factor analysis was conducted on all measurement items. The results revealed multiple factors, with the first factor accounting for 26.67% of the variance, which is well below the critical threshold of 40% (Tang and Wen, 2020). These findings suggest that common method bias is not a significant concern in this study.

### 3.2 Descriptive statistics and correlation analysis

Table 2 presents the means, standard deviations, and Pearson correlation matrix for the study variables. Correlation analysis revealed significant relationships among exercise adherence, exercise commitment, mindfulness, psychological resilience, negative mental health factors, life satisfaction, positive affect, and negative affect, which align with theoretical expectations. These results provide preliminary support for the subsequent mediation analysis. Additionally, demographic variables such as gender, birthplace, and only-child status showed significant correlations with certain key variables, highlighting their potential role as confounding factors. As a result, these variables were included as control variables in the mediation analysis. Furthermore, previous research indicates that BMI is significantly associated with both physical exercise and mental health outcomes (Eddolls et al., 2018; Herhaus et al., 2020). Consequently, BMI was incorporated into the model to ensure the validity of the findings.

**Table 2:** Descriptive statistics and Pearson’s correlations between the study variables.

Variables	1	2	3	4	5	6	7	8	9	10	11	12	13	14
1. Gender	-													
2. Age	0.04	-												
3. Grade	0.00	0.81**	-											
4. Birthplace	−0.04	−0.02	−0.01	-										
5. Only-child status	0.08*	0.02	0.00	0.38**	-									
6. BMI	0.18**	−0.02	−0.03	0.03	0.06	-								
7. EA	0.24**	−0.04	−0.05	−0.01	−0.01	0.01	-							
8. EC	0.20**	−0.03	−0.04	0.02	0.00	−0.04	0.78**	-						
9. NMHF	0.00	0.02	0.00	−0.10**	−0.05	0.12**	−0.28**	−0.36**	-					
10. Mindful	−0.12**	−0.02	0.01	0.11**	0.07*	−0.07	0.23**	0.27**	−0.48**	-				
11. Resilience	0.05	−0.02	−0.04	0.11**	0.03	−0.02	0.23**	0.28**	−0.57**	0.36**	-			
12. PMHF	0.01	−0.07	−0.05	0.13**	0.08*	−0.06	0.33**	0.37**	−0.54**	0.33**	0.37**	-		
13. PA	−0.01	−0.02	0.04	0.10**	0.07	−0.03	0.30**	0.38**	−0.56**	0.39**	0.46**	0.57**	-	
14. NA	0.08*	0.03	0.01	−0.08*	−0.02	0.04	−0.13**	−0.11**	0.43**	−0.38**	−0.38**	−0.24**	−0.23**	-
M	1.53	19.78	2.43	1.61	1.59	23.30	3.34	3.43	1.02	4.05	3.34	4.21	3.30	2.22
SD	0.50	1.31	1.09	0.49	0.49	6.76	0.86	0.83	0.47	0.80	0.57	1.08	0.83	0.80

Gender: male = 1, female = 2; Birthplace: urban = 1, rural = 2; Only-child status: yes = 1, no = 2; BMI, body mass index; EA, exercise adherence; EC, exercise commitment; NMHF, negative mental health factors; PMHF, positive mental health factors; PA, positive affect; NA, negative affect; * *p* < 0.05, ** *p* < 0.01.

### 3.3 Structural model construction and testing

Mplus 8.3 was employed to evaluate the fit between the measurement model and the observed data. The results indicated that the model met the ideal fit criteria: *x*^2^/*df* = 1.62, RMSEA = 0.03, CFI = 0.99, TLI = 0.99, and SRMR = 0.02, supporting further structural model testing. To address potential multicollinearity among the independent variables, the variance inflation factor (VIF) was calculated using the formula *VIF_j_* = 1/(1-*R_j_*^2^), where *R_j_*^2^ represents the coefficient of determination for the regression of one independent variable on the others. All *VIF* values for the predictor variables were below 5, indicating that multicollinearity was not a concern (Wen et al., 2018).

### 3.4 Mediation effect testing

**Figure 2:**
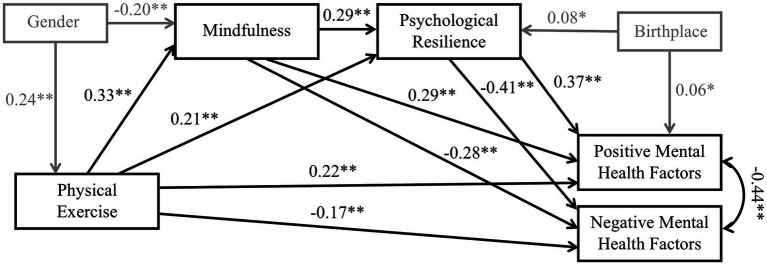
Statistical model with standardized path coefficients. Control variables and their significant paths are displayed in gray; non-significant paths of the control variables are not displayed. * *p* < 0.05 ** *p* < 0.01.

Mediation analysis of the constructed model was conducted with gender, birthplace, only-child status, and BMI as control variables. All paths between the main variables were significant and consistent with theoretical predictions. Among the control variables, gender was significantly positively associated with physical exercise (*β* = 0.24, *p* < 0.01) and negatively associated with mindfulness (*β* = −0.20, *p* < 0.01). Birthplace was significantly positively associated with resilience (*β* = 0.08, *p* < 0.05) and subjective well-being (*β* = 0.06, *p* < 0.05). The final model with standardized path estimates is shown in Figure 2. All standardized and unstandardized regression coefficients for the direct and indirect effects of the model are listed in Tables 3, 4.

**Table 3:** Direct effects of the path model.

Variable	*B*	SE	*p*	95% CI	*β*	SE	*p*	95% CI	*R* ^2^
Positive mental health factors (Y1)									0.437
Resilience	0.37	0.04	<0.01	[0.28, 0.45]	0.37	0.05	<0.01	[0.28, 0.45]	
Mindfulness	0.29	0.05	<0.01	[0.21, 0.38]	0.29	0.04	<0.01	[0.21, 0.38]	
Physical exercise	0.26	0.05	<0.01	[0.17, 0.35]	0.22	0.04	<0.01	[0.15, 0.29]	
Birthplace	0.13	0.06	0.03	[0.01, 0.24]	0.06	0.03	0.03	[0.00, 0.12]	
Negative mental health factors (Y2)									0.436
Resilience	−0.41	0.04	<0.01	[−0.48, −0.34]	−0.41	0.04	<0.01	[−0.49, −0.34]	
Mindfulness	−0.28	0.04	<0.01	[−0.35, −0.20]	−0.28	0.04	<0.01	[−0.35, −0.20]	
Physical exercise	−0.20	0.05	<0.01	[−0.30, −0.10]	−0.17	0.04	<0.01	[−0.25, −0.09]	
Birthplace	−0.05	0.06	0.35	[−0.17, 0.05]	−0.03	0.03	0.35	[−0.08, 0.03]	
BMI	0.08	0.04	0.05	[0.01, 0.17]	0.08	0.04	0.06	[0.01, 0.17]	
Mindfulness (M1)									0.132
Physical exercise	0.40	0.06	<0.01	[0.29, 0.51]	0.33	0.04	<0.01	[0.25, 0.42]	
Only-child Status	0.13	0.08	0.10	[−0.02, 0.28]	0.06	0.04	0.10	[−0.01, 0.14]	
Gender	−0.4	0.07	<0.01	[−0.55, −0.26]	−0.20	0.04	<0.01	[−0.27, −0.13]	
Birthplace	0.15	0.08	0.06	[−0.01, 0.30]	0.07	0.04	0.06	[−0.01, 0.15]	
Resilience (M2)									0.174
Physical exercise	0.25	0.06	<0.01	[0.15, 0.36]	0.21	0.04	<0.01	[0.13, 0.30]	
Mindfulness	0.29	0.05	<0.01	[0.20, 0.38]	0.29	0.05	<0.01	[0.19, 0.38]	
Birthplace	0.16	0.07	0.02	[0.03, 0.31]	0.08	0.04	0.02	[0.01, 0.15]	
Physical exercise (X)									
Gender	0.40	0.08	<0.01	[0.25, 0.55]	0.24	0.04	<0.01	[0.15, 0.31]	

For clarity and precision, R^2^ values are reported to three decimal places, while other values are reported to two decimal places.

**Table 4:** Indirect effects of the path model.

Variable	*B*	SE	*p*	95% CI	*β*	SE	*p*	95% CI	% of Total
Positive mental health factors (Y1)									
Total effect	0.51	0.05	<0.01	[0.40, 0.61]	0.43	0.04	<0.01	[0.35, 0.50]	100.00%
Total indirect effect	0.25	0.03	<0.01	[0.19, 0.31]	0.21	0.02	<0.01	[0.16, 0.26]	48.84%
X → M1 → Y1	0.11	0.03	<0.01	[0.07, 0.17]	0.10	0.02	<0.01	[0.06, 0.14]	23.26%
X → M2 → Y1	0.09	0.03	<0.01	[0.05, 0.15]	0.08	0.02	<0.01	[0.04, 0.12]	18.60%
X → M1 → M2 → Y1	0.04	0.01	<0.01	[0.03, 0.07]	0.04	0.01	<0.01	[0.02, 0.06]	9.30%
Negative mental health factors (Y2)									
Total effect	−0.46	0.06	<0.01	[−0.58, −0.34]	−0.39	0.04	<0.01	[−0.47, −0.31]	100.00%
Total indirect effect	−0.26	0.03	<0.01	[−0.33, −0.20]	−0.22	0.03	<0.01	[−0.27, −0.17]	56.41%
X → M1 → Y2	−0.11	0.02	<0.01	[−0.16, −0.07]	−0.09	0.02	<0.01	[−0.13, −0.06]	23.08%
X → M2 → Y2	−0.10	0.03	<0.01	[−0.16, −0.06]	−0.09	0.02	<0.01	[−0.13, −0.05]	23.08%
X → M1 → M2 → Y2	−0.05	0.01	<0.01	[−0.07, −0.03]	−0.04	0.01	<0.01	[−0.06, −0.02]	10.26%

X = Physical exercise; M1 = Mindful; M2 = Psychological resilience.

As shown in Table 3, the direct effect analysis indicated that, after controlling for the influence of control variables, physical exercise (*β* = 0.22, *p* < 0.01), mindfulness (*β* = 0.29, *p* < 0.01), and psychological resilience (*β* = 0.37, *p* < 0.01) exerted significant positive effects on positive mental health factors, collectively explaining 43.7% of the variance. In contrast, physical exercise (*β* = −0.17, *p* < 0.01), mindfulness (*β* = −0.28, *p* < 0.01), and psychological resilience (*β* = −0.41, *p* < 0.01) exhibited significant negative effects on negative mental health factors, accounting for 43.6% of the variance. These findings highlight the pivotal roles of physical exercise, mindfulness, and psychological resilience in shaping both positive and negative dimensions of mental health.

As shown in Table 4, mindfulness and psychological resilience significantly mediated the effects of physical exercise on both positive and negative mental health factors. For positive mental health factors (Y1), physical exercise exhibited a total effect of *β* = 0.43 (*p* < 0.01, 95% CI: [0.35, 0.50]). Of this total effect, 48.84% was mediated through mindfulness (*β* = 0.10, *p* < 0.01), psychological resilience (*β* = 0.08, *p* < 0.01), and their chain mediation (*β* = 0.04, *p* < 0.01). For negative mental health factors (Y2), physical exercise demonstrated a total effect of *β* = −0.39 (*p* < 0.01, 95% CI: [−0.47, −0.31]), with 56.41% mediated through mindfulness (*β* = −0.09, *p* < 0.01), psychological resilience (*β* = −0.09, *p* < 0.01), and their chain mediation (*β* = −0.04, *p* < 0.01).

Despite the relatively low absolute values of path coefficients and mediation effects in our model (*β* < 0.4, mediation *β* < 0.1), all effects were statistically significant (*p* < 0.01), and the overall model explained approximately 43% of the variance, suggesting that the relationships between variables are stable and practically meaningful (MacKinnon et al., 2002; Preacher and Hayes, 2008).

## 4 Discussion

Given the severity of mental health issues among university students, this study developed a model based on the COR theory and the DFM to further elucidate the mechanisms underlying the impact of physical exercise on mental health. Specifically, the mediating roles of mindfulness and psychological resilience in the relationship between physical exercise and mental health were examined. Our findings provide valuable insights into the complex relationships among physical exercise, mindfulness, psychological resilience, and mental health.

### 4.1 The relationship between physical exercise and mental health

This study demonstrates that physical exercise has a significant positive impact on enhancing positive mental health factors among university students while playing a critical role in mitigating negative mental health factors. This finding aligns closely with the fundamental assumptions of the COR theory. COR theory posits that individuals acquire, maintain, and protect valuable resources (e.g., psychological resources) to better cope with life stressors and challenges (Hobfoll, 1989; Hobfoll, 2001). University students undergoing a transitional stage of life often face academic pressure, interpersonal challenges, and career uncertainty, which can result in the continuous depletion of psychological resources (Wu et al., 2020). As an active and effective resource investment behavior, physical exercise helps students accumulate both physiological and psychological resources in the process of coping with stress. Specifically, engaging in physical exercise can directly enhance positive emotional experiences and reduce negative emotional states (e.g., anxiety and depression) (Mahindru et al., 2023). This not only helps prevent resource depletion but also fosters a gain spiral. Therefore, from the perspective of COR theory, the findings of this study highlight the critical role of physical exercise as a proactive resource investment behavior in safeguarding university students’ mental health.

### 4.2 The mediating role of mindfulness and psychological resilience

This study underscores the critical mediating roles of mindfulness and psychological resilience in the relationship between physical exercise and mental health among university students. Specifically, physical exercise significantly enhances students’ mindfulness and resilience levels, which, in turn, improve positive mental health factors while reducing negative ones.

#### 4.2.1 Mindfulness as a mediator

This study provides new insights by emphasizing the role of mindfulness as a key mediating variable in the relationship between physical exercise and university students’ mental health. Although prior studies have highlighted the benefits of physical exercise in reducing anxiety and depression and enhancing well-being among university students (Herbert, 2022; Huang et al., 2024), limited attention has been given to the mediating role of mindfulness in this process. This study provides robust evidence that mindfulness not only a psychological resource cultivated through physical exercise but also a key mediating mechanism linking exercise to the improvement of university students’ mental health. According to COR theory, mindfulness is a critical personal psychological resource that enhances individuals’ present-moment awareness and nonjudgmental attention, enabling them to manage emotions and thoughts more effectively in stressful situations while reducing resource loss (Kabat-Zinn, 2003; Brown et al., 2007), thereby improving mental health. The process of physical exercise often involves heightened bodily awareness and attention to the present moment, particularly in practices like Tai Chi and Pilates (Caldwell et al., 2010). Therefore, consistent engagement in physical exercise can significantly enhance university students’ mindfulness levels, fostering the development and reinforcement of this essential internal resource. These findings broaden existing theoretical frameworks on the link between physical exercise and mindfulness, offering a theoretical basis for understanding how physical exercise supports the development of internal psychological resources and mental health.

#### 4.2.2 Resilience as a mediator

This study found that psychological resilience emerges as a significant mediator in the relationship between physical exercise and university students’ mental health, aligning with findings from latest meta-analytic structural equation modeling studies (Lin et al., 2024). Psychological resilience is a key psychological resource that facilitates individuals’ adaptation and recovery from adversity (Southwick et al., 2014). According to the COR theory, psychological resilience effectively protects existing resources and promotes resource accumulation, enabling individuals to better cope with environmental challenges, manage stress more efficiently, regulate emotions, and maintain mental well-being (Mubarak et al., 2022; Siddiquei et al., 2025). Furthermore, the competitive, open, and dynamic nature of physical exercise exposes individuals to moderate psychological challenges, allowing them to develop adaptive coping strategies and regain physiological and psychological stability following stress exposure (Li, 2017). This repeated stress-recovery cycle serves as a training ground for resource accumulation, progressively strengthening psychological resilience and enhancing individuals’ ability to protect and mobilize resources to navigate future challenges. Thus, psychological resilience serves as a critical mediator between physical exercise and mental health, not only transforming the positive effects of physical exercise into psychological resource accumulation but also fostering long-term psychological adaptation and overall mental well-being.

### 4.3 The chain mediation role of mindfulness and resilience

One significant contribution of this study is the identification of mindfulness and resilience as sequential mediation mechanism, forming a resource chain that links physical exercise to the improvement of university students’ mental health. This provides new insights into the overall process through which physical exercise promotes mental well-being. According to the COR theory, psychological resources do not exist in isolation but are interconnected, forming resource caravans that collectively function as an integrated system (Hobfoll, 2011). The sequential mediation effect identified in this study reflects this resource caravan effect, highlighting the systematic accumulation and interactive nature of psychological resources.

To begin with, physical exercise facilitates the development of mindfulness as a psychological resource, enhancing individuals’ awareness and regulation of their emotions and stress. The accumulation of this resource helps individuals more effectively manage stress and regulate their emotional responses. It not only mitigates automatic and maladaptive reactions to current frustrations and stressors (Arpaci and Gundogan, 2020) but also prevents the development of rumination and depressive thinking, thereby enhancing individuals’ resilience (Thompson et al., 2011; Bajaj and Pande, 2016).

Building upon this foundation, enhanced psychological resilience further strengthens individuals’ capacity to recover from adversity and their ability to mobilize and utilize resources effectively. This sequential process exemplifies the resource gain spiral described in COR theory, wherein the accumulation of specific psychological resources (e.g., increased mindfulness) facilitates the development of additional resources (e.g., psychological resilience), enhancing individuals’ adaptive capacity in stressful situations. This mechanism enables university students to navigate academic, social, and career-related challenges more efficiently, ultimately contributing to improved overall mental health (Hobfoll, 2011).

Thus, this study reveals the synergistic mediating role of mindfulness and psychological resilience in the relationship between physical exercise and mental health, elucidating the hierarchical accumulation of psychological resources within the resource gain spiral and expanding the mechanistic understanding of how physical exercise fosters mental well-being.

### 4.4 Theoretical implications

The theoretical contributions of this study are mainly reflected in the following three aspects:

Firstly, it deepens the theoretical application of the DFM of mental health. Traditional studies have often focused on the impact of physical exercise on a single dimension of mental health, such as reducing negative symptoms or enhancing well-being. However, by incorporating the DFM, this study considers both positive and negative aspects of mental health, demonstrating the comprehensive influence of physical exercise on mental health through the enhancement of mindfulness and resilience. The findings not only support the applicability of the DFM in the university student population but also provide a new theoretical perspective for exploring the complex mechanisms of mental health in future research.

Secondly, it reveals the multidimensional chain mechanism between physical exercise and mental health. Based on COR theory, this study elucidates how physical exercise enhances university students’ mental health through the sequential pathway of mindfulness and psychological resilience. This provides a brand-new theoretical perspective in the field of exercise psychology. This contributes to the theoretical foundation for developing multidimensional interventions, underscoring the importance of integrating mindfulness and resilience training into exercise-based mental health programs.

Finally, this study expands the applicability and theoretical scope of the COR theory in mental health research. For the first time, it systematically applies COR theory to explain the complex multivariable interactions among physical exercise, mindfulness, psychological resilience, and mental health. This study enriches the theoretical perspective of COR theory regarding resource accumulation, conservation, and the gain spiral effect. Furthermore, it proposes that mindfulness and psychological resilience, as key psychological resources, not only independently mitigate resource depletion caused by stress but also function synergistically. This highlights the integrative nature and explanatory power of COR theory, providing a foundation for further theoretical advancements and empirical exploration.

### 4.5 Practical implications

From a practical perspective, this study provides valuable insights and actionable strategies for improving mental health interventions among university students. First, physical exercise, as a simple yet effective intervention, has been shown to significantly enhance mental health through both direct effects and mediating mechanisms, such as mindfulness and resilience. Promoting physical exercise as part of routine mental health management in settings such as universities and workplaces could effectively alleviate stress, reduce negative emotions, and enhance well-being.

Second, this study highlights the critical roles of mindfulness and resilience in mental health, providing a theoretical foundation for interventions that integrate mindfulness and resilience training. In recent years, Mindfulness-Based Stress Reduction (MBSR) has been widely applied to reduce stress and improve mental health (Khoury et al., 2015). Our findings further demonstrate that mindfulness not only directly benefits mental health but also indirectly enhances it by strengthening resilience. Therefore, combining mindfulness training with resilience-enhancing activities, such as Cognitive Behavioral Therapy (CBT) or stress management courses, could create more comprehensive and systematic mental health intervention programs.

Finally, the discovery of the chain mediating effect suggests that mindfulness and resilience synergistically amplify the positive impact of physical exercise on mental health. Based on this finding, designing multidimensional intervention programs that integrate physical exercise, mindfulness training, and resilience enhancement can maximize improvements in mental health outcomes. This holistic, multidimensional approach is not only applicable to university students but can also be extended to other populations, such as workplace employees and older adults. It offers an effective pathway for managing life stressors and promoting overall mental well-being.

### 4.6 Limitations and future directions

The limitations of this study are as follows. First, the cross-sectional design restricts the ability to establish causal relationships among physical exercise, mindfulness, resilience, and mental health. While the findings highlight significant associations and mediation effects, the temporal sequence of these variables remains unclear. Future research should consider longitudinal or experimental designs to clarify causality and capture dynamic interactions over time, thereby enhancing the robustness of these conclusions.

Second, the reliance on self-reported data introduces the potential for social desirability bias, which may lead to overestimation or underestimation of the observed effects. Incorporating physiological measures or third-party assessments in future studies could improve the objectivity and reliability of the data.

Finally, the sample was limited to Chinese university students, which may constrain the generalizability of the findings. Expanding the sample to include students from diverse cultural and regional backgrounds in future research would enhance the applicability and cross-cultural validity of the results.

## 5 Conclusion

This study highlights the critical roles of physical exercise, mindfulness, and psychological resilience in promoting mental health among university students. Grounded in the COR theory and the DFM, our findings systematically demonstrate that physical exercise, as a proactive resource investment behavior, has significant effects on both positive and negative dimensions of mental health, with mindfulness and resilience serving as key mediators in this relationship. Importantly, the sequential mediating effect of mindfulness and resilience reveals the resource gain spiral and resource caravan effect described in COR theory. This contributes to a more comprehensive understanding of the mechanisms through which physical exercise enhances mental health. These insights provide theoretical and practical implications for developing comprehensive mental health interventions.

## Data Availability

The raw data supporting the conclusions of this article will be made available by the authors, without undue reservation.

## Footnotes

^1^The DSM-IV/CIDI disorder refers to mental health conditions that are diagnosed based on the criteria established in the Diagnostic and Statistical Manual of Mental Disorders, Fourth Edition (DSM-IV), and assessed using the Composite International Diagnostic Interview (CIDI).

## References

[ref2] Antaramian S. (2015). Assessing psychological symptoms and well-being: application of a dual-factor mental health model to understand college student performance. J. Psychoeduc. Assess. 33, 419–429. doi: 10.1177/0734282914557727

[ref3] Antaramian S. P. Huebner E. S. Hills K. J. Valois R. F. (2010). A dual-factor model of mental health: toward a more comprehensive understanding of youth functioning. Am. J. Orthopsychiatry 80, 462–472. doi: 10.1111/j.1939-0025.2010.01049.x, PMID: 20950287

[ref4] Antonini Philippe R. Schwab L. Biasutti M. (2021). Effects of physical activity and mindfulness on resilience and depression during the first wave of COVID-19 pandemic. Front. Psychol. 12:742. doi: 10.3389/fpsyg.2021.700742, PMID: 34393936 PMC8360111

[ref5] Arpaci I. Gundogan S. (2020). Mediating role of psychological resilience in the relationship between mindfulness and nomophobia. Br. J. Guid. Couns. 50, 782–790. doi: 10.1080/03069885.2020.1856330, PMID: 40101104

[ref6] Auerbach R. P. Alonso J. Axinn W. G. Cuijpers P. Ebert D. D. Green J. G.. (2016). Mental disorders among college students in the World Health Organization world mental health surveys. Psychol. Med. 46, 2955–2970. doi: 10.1017/S0033291716001665, PMID: 27484622 PMC5129654

[ref7] Bajaj B. Pande N. (2016). Mediating role of resilience in the impact of mindfulness on life satisfaction and affect as indices of subjective well-being. Personal. Individ. Differ. 93, 63–67. doi: 10.1016/j.paid.2015.09.005

[ref8] Bieda A. Hirschfeld G. Schönfeld P. Brailovskaia J. Lin M. Margraf J. (2019). Happiness, life satisfaction and positive mental health: investigating reciprocal effects over four years in a Chinese student sample. J. Res. Pers. 78, 198–209. doi: 10.1016/j.jrp.2018.11.012

[ref9] Brown K. W. Ryan R. M. (2003). The benefits of being present: mindfulness and its role in psychological well-being. J. Pers. Soc. Psychol. 84, 822–848. doi: 10.1037/0022-3514.84.4.822 12703651

[ref10] Brown K. W. Ryan R. M. Creswell J. D. (2007). Mindfulness: theoretical foundations and evidence for its salutary effects. Psychol. Inq. 18, 211–237. doi: 10.1080/10478400701598298

[ref11] Caldwell K. Harrison M. Adams M. Quin R. H. Greeson J. (2010). Developing mindfulness in college students through movement-based courses: effects on self-regulatory self-efficacy, mood, stress, and sleep quality. J. Am. Coll. Heal. 58, 433–442. doi: 10.1080/07448480903540481, PMID: 20304755 PMC2879280

[ref12] Chen S. Y. Cui H. Zhou R. L. Jia Y. Y. (2012). Revision of mindful attention awareness scale (MAAS). Chin. J. Clin. Psychol. 20, 148–151. doi: 10.16128/j.cnki.1005-3611.2012.02.024

[ref13] Chen W. Liu J. Luo J. Liu G. Q. (2020). Reliability and validity of the Chinese version of brief resilience scale. Chin. J. Clin. Psych. 28, 24–28. doi: 10.16128/j.cnki.1005-3611.2020.01.006

[ref15] Cuijpers P. Auerbach R. P. Benjet C. Bruffaerts R. Ebert D. Karyotaki E.. (2019). The world health organization world mental health international college student initiative: an overview. Int. J. Methods Psychiatr. Res. 28:e1761. doi: 10.1002/mpr.1761, PMID: 30614123 PMC6590455

[ref16] Davydov D. M. Stewart R. Ritchie K. Chaudieu I. (2010). Resilience and mental health. Clin. Psychol. Rev. 30, 479–495. doi: 10.1016/j.cpr.2010.03.003, PMID: 20395025

[ref17] Diener E. D. Emmons R. A. Larsen R. J. Griffin S. (1985). The satisfaction with life scale. J. Pers. Assess. 49, 71–75. doi: 10.1207/s15327752jpa4901_13, PMID: 16367493

[ref18] Doré I. Sylvester B. Sabiston C. Sylvestre M.-P. O. Loughlin J. Brunet J.. (2020). Mechanisms underpinning the association between physical activity and mental health in adolescence: a 6-year study. Int. J. Behav. Nutr. Phys. Act. 17:9. doi: 10.1186/s12966-020-0911-5, PMID: 32005251 PMC6993479

[ref19] Dunston E. R. Messina E. S. Coelho A. J. Chriest S. N. Waldrip M. P. Vahk A.. (2020). Physical activity is associated with grit and resilience in college students: is intensity the key to success? J. Am. Coll. Heal. 70, 216–222. doi: 10.1080/07448481.2020.1740229, PMID: 32240056

[ref20] Dvořáková K. Kishida M. Li J. Elavsky S. Broderick P. C. Agrusti M. R.. (2017). Promoting healthy transition to college through mindfulness training with first-year college students: pilot randomized controlled trial. J. Am. Coll. Heal. 65, 259–267. doi: 10.1080/07448481.2017.1278605, PMID: 28076182 PMC5810370

[ref21] Eddolls W. T. B. McNarry M. A. Lester L. Winn C. O. N. Stratton G. Mackintosh K. A. (2018). The association between physical activity, fitness and body mass index on mental well-being and quality of life in adolescents. Qual. Life Res. 27, 2313–2320. doi: 10.1007/s11136-018-1915-3, PMID: 29948603 PMC6132966

[ref22] Egozi Farkash H. Lahad M. Hobfoll S. E. Leykin D. Aharonson-Daniel L. (2022). Conservation of resources, psychological distress, and resilience during the COVID-19 pandemic. Int. J. Public Health 67:1604567. doi: 10.3389/ijph.2022.1604567, PMID: 36119444 PMC9472268

[ref23] Fang J. Zhang M. Q. (2012). Assessing point and interval estimation for the mediating effect: distribution of theProduct, nonparametric bootstrap and Markov chain Monte Carlo methods. Acta Psychol. Sin. 44, 1408–1420. doi: 10.3724/SP.J.1041.2012.01408, PMID: 37113526

[ref25] Fung S. F. (2020). Validity of the brief resilience scale and brief resilient coping scale in a Chinese sample. Int. J. Environ. Res. Public Health 17:1265. doi: 10.3390/ijerph17041265, PMID: 32079115 PMC7068432

[ref26] González-Martín A. M. Aibar-Almazán A. Rivas-Campo Y. Castellote-Caballero Y. Carcelén-Fraile M. D. C. (2023). Mindfulness to improve the mental health of university students. A systematic review and meta-analysis. Front. Public Health 11:1284632. doi: 10.3389/fpubh.2023.1284632, PMID: 38111480 PMC10726040

[ref27] Greenspoon P. J. Saklofske D. H. (2001). Toward an integration of subjective well-being and psychopathology. Soc. Indic. Res. 54, 81–108. doi: 10.1023/a:1007219227883

[ref28] Herbert C. (2022). Enhancing mental health, well-being and active lifestyles of university students by means of physical activity and exercise research programs. Front. Public Health 10:849093. doi: 10.3389/fpubh.2022.849093, PMID: 35548074 PMC9082407

[ref29] Herhaus B. Kersting A. Brähler E. Petrowski K. (2020). Depression, anxiety and health status across different BMI classes: a representative study in Germany. J. Affect. Disord. 276, 45–52. doi: 10.1016/j.jad.2020.07.020, PMID: 32697715

[ref30] Hobfoll S. E. (1989). Conservation of resources: a new attempt at conceptualizing stress. Am. Psychol. 44, 513–524. doi: 10.1037/0003-066X.44.3.513, PMID: 2648906

[ref31] Hobfoll S. E. (2001). The influence of culture, community, and the nested-self in the stress process: advancing conservation of resources theory. Appl. Psychol. 50, 337–421. doi: 10.1111/1464-0597.00062, PMID: 40143556

[ref32] Hobfoll S. E. (2011). Conservation of resource caravans and engaged settings. J. Occup. Organ. Psychol. 84, 116–122. doi: 10.1111/j.2044-8325.2010.02016.x

[ref33] Hu T. Q. Zhang D. J. Wang J. L. (2015). A meta-analysis of the trait resilience and mental health. Personal. Individ. Differ. 76, 18–27. doi: 10.1016/j.paid.2014.11.039, PMID: 40157298

[ref34] Huang K. Beckman E. M. Ng N. Dingle G. A. Han R. James K.. (2024). Effectiveness of physical activity interventions on undergraduate students’ mental health: systematic review and meta-analysis. Health Promot. Int. 39:daae054. doi: 10.1093/heapro/daae054, PMID: 38916148 PMC11196957

[ref35] Jackson D. L. Gillaspy J. A. Jr. Purc-Stephenson R. (2009). Reporting practices in confirmatory factor analysis: an overview and some recommendations. Psychol. Methods 14, 6–23. doi: 10.1037/a0014694, PMID: 19271845

[ref36] Jiang Y. M. Ding C. Shen B. (2023). Latent profile analysis of mental health among Chinese university students: evidence for the dual-factor model. Healthcare 11:2719. doi: 10.3390/healthcare11202719, PMID: 37893793 PMC10606236

[ref37] Jiang Y. Zhang L. W. Mao Z. X. (2018). Physical exercise and mental health: the effect of emotion Regulation Self-efficacy and emotion regulation strategy. Stud. Psychol. Behav. 16, 570–576.

[ref38] Jin Y. C. Zhang M. Y. Wang Y. N. An J. X. (2020). The relationship between trait mindfulness, loneliness, regulatory emotional self-efficacy, and subjective well-being. Personal. Individ. Differ. 154:109650. doi: 10.1016/j.paid.2019.109650

[ref39] Kabat-Zinn J. (2003). Mindfulness-based interventions in context: past, present, and future. Clin. Psychol. Sci. Pract. 10, 144–156. doi: 10.1093/clipsy.bpg016

[ref40] Kettunen O. Vuorimaa T. Vasankari T. (2015). A 12-month exercise intervention decreased stress symptoms and increased mental resources among working adults–results perceived after a 12-month follow-up. Int. J. Occup. Med. Environ. Health 28, 157–168. doi: 10.13075/ijomeh.1896.00263, PMID: 26159956

[ref41] Keyes C. L. M. (2002). The mental health continuum: from languishing to flourishing in life. J. Health Soc. Behav. 43:207. doi: 10.2307/3090197, PMID: 12096700

[ref42] Keyes C. L. M. (2005). Mental illness and/or mental health? Investigating axioms of the complete state model of health. J. Consult. Clin. Psychol. 73, 539–548. doi: 10.1037/0022-006X.73.3.539, PMID: 15982151

[ref43] Khoury B. Sharma M. Rush S. E. Fournier C. (2015). Mindfulness-based stress reduction for healthy individuals: a meta-analysis. J. Psychosom. Res. 78, 519–528. doi: 10.1016/j.jpsychores.2015.03.009, PMID: 25818837

[ref44] Li J. C. (2017). Exercise psychology. Beijing: Higher Education Press.

[ref45] Li Y. Wang A. W. Wu Y. L. Han N. N. Huang H. M. (2021). Impact of the COVID-19 pandemic on the mental health of college students: a systematic review and meta-analysis. Front. Psychol. 12:669119. doi: 10.3389/fpsyg.2021.669119, PMID: 34335381 PMC8316976

[ref46] Li X. N. Yu H. Yang N. (2021). The mediating role of resilience in the effects of physical exercise on college students’ negative emotions during the COVID-19 epidemic. Sci. Rep. 11:24510. doi: 10.1038/s41598-021-04336-y, PMID: 34972833 PMC8720086

[ref47] Lin H. Zhu Y. Y. Liu Q. Z. Li S. (2024). The mediating effect of resilience between physical activity and mental health: a meta-analytic structural equation modeling approach. Front. Public Health 12:1434624. doi: 10.3389/fpubh.2024.1434624, PMID: 39411497 PMC11473373

[ref48] Liu X. Q. Ping S. Q. Gao W. J. (2019). Changes in undergraduate students’ psychological well-being as they experience university life. Int. J. Environ. Res. Public Health 16:2864. doi: 10.3390/ijerph16162864, PMID: 31405114 PMC6719208

[ref49] MacKinnon D. P. Lockwood C. M. Hoffman J. M. West S. G. Sheets V. (2002). A comparison of methods to test mediation and other intervening variable effects. Psychol. Methods 7, 83–104. doi: 10.1037/1082-989X.7.1.83, PMID: 11928892 PMC2819363

[ref50] Mahindru A. Patil P. Agrawal V. (2023). Role of physical activity on mental health and well-being: a review. Cureus 15:e33475. doi: 10.7759/cureus.33475, PMID: 36756008 PMC9902068

[ref51] Makowska Z. Merecz D. (2000). The usefulness of the health status questionnaire: D. Goldberg's GHQ-12 and GHQ-28 for diagnosis of mental disorders in workers. Med. Pr. 51, 589–601. 11288687

[ref52] Mikkelsen K. Stojanovska L. Polenakovic M. Bosevski M. Apostolopoulos V. (2017). Exercise and mental health. Maturitas 106, 48–56. doi: 10.1016/j.maturitas.2017.09.003 29150166

[ref53] Mubarak N. Khan J. Khan A. K. (2022). Psychological distress and project success: the moderating role of employees’ resilience and mindfulness. Int. J. Proj. Manag. 40, 566–576. doi: 10.1016/j.ijproman.2022.05.004, PMID: 40157298

[ref54] Peng Y. Q. Ju M. Z. (2013). The “heart” of the working mechanism of mindfulness: attention or attitude? J. Psychol. Sci. 36, 1009–1013. doi: 10.16719/j.cnki.1671-6981.2013.04.042

[ref55] Podsakoff P. M. MacKenzie S. B. Lee J.-Y. Podsakoff N. P. (2003). Common method biases in behavioral research: a critical review of the literature and recommended remedies. J. Appl. Psychol. 88, 879–903. doi: 10.1037/0021-9010.88.5.879, PMID: 14516251

[ref56] Preacher K. J. Hayes A. F. (2008). Asymptotic and resampling strategies for assessing and comparing indirect effects in multiple mediator models. Behav. Res. Methods 40, 879–891. doi: 10.3758/BRM.40.3.879, PMID: 18697684

[ref57] Qiu L. Zheng X. Wang Y. F. (2008). Revision of the positive affect and negative affect scale. Appl. Psychol. 14, 249–268.

[ref58] Román-Mata S. Puertas-Molero P. Ubago-Jiménez J. L. González-Valero G. (2020). Benefits of physical activity and its associations with resilience, emotional intelligence, and psychological distress in university students from southern Spain. Int. J. Environ. Res. Public Health 17:4474. doi: 10.3390/ijerph17124474, PMID: 32580322 PMC7344387

[ref59] Romppel M. Braehler E. Roth M. Glaesmer H. (2013). What is the general health Questionnaire-12 assessing?: dimensionality and psychometric properties of the general health Questionnaire-12 in a large scale German population sample. Compr. Psychiatry 54, 406–413. doi: 10.1016/j.comppsych.2012.10.010, PMID: 23206494

[ref60] Russell V. A. Zigmond M. J. Dimatelis J. J. Daniels W. M. U. Mabandla M. V. (2014). The interaction between stress and exercise, and its impact on brain function. Metab. Brain Dis. 29, 255–260. doi: 10.1007/s11011-013-9479-y 24399497

[ref61] Shu M. L. Liao X. Y. Qin L. L. (2021). Mental health and its influencing factors of college students in Chang sha city in the “post-EpidemicEra”. Chin. J. Health Psychol. 29, 1712–1717. doi: 10.13342/j.cnki.cjhp.2021.11.024

[ref62] Siddiquei A. N. Ahmad S. Asmi F. (2025). Fostering team resilience with servant leadership: a multi-level study of the construction industry. Acta Psychol. 253:104732. doi: 10.1016/j.actpsy.2025.104732, PMID: 39893795

[ref63] Smith B. W. Dalen J. Wiggins K. Tooley E. Christopher P. Bernard J. (2008). The brief resilience scale: assessing the ability to bounce back. Int. J. Behav. Med. 15, 194–200. doi: 10.1080/10705500802222972, PMID: 18696313

[ref64] Southwick S. M. Bonanno G. A. Masten A. S. Panter-Brick C. Yehuda R. (2014). Resilience definitions, theory, and challenges: interdisciplinary perspectives. Eur. J. Psychotraumatol. 5:25338. doi: 10.3402/ejpt.v5.25338, PMID: 25317257 PMC4185134

[ref65] Suldo S. M. Shaffer E. J. (2008). Looking beyond psychopathology: the dual-factor model of mental health in youth. Sch. Psychol. Rev. 37, 52–68. doi: 10.1080/02796015.2008.12087908

[ref66] Tang H. Y. Chen Q. Wu J. H. (2021). The relationship between college Student’s social development Level and mental health: the mediating role of alexithymia and the gender difference. Psychol. Dev. Educ. 37, 735–742. doi: 10.16187/j.cnki.issn1001-4918.2021.05.15

[ref67] Tang D. D. Wen Z. L. (2020). Statistical approaches for testing common method Bias: problems and suggestions. J. Psychol. Sci. 43, 215–223. doi: 10.16719/j.cnki.1671-6981.20200130

[ref68] Taylor A. B. MacKinnon D. P. Tein J.-Y. (2007). Tests of the three-path mediated effect. Organ. Res. Methods 11, 241–269. doi: 10.1177/1094428107300344, PMID: 40144045

[ref69] Thompson R. W. Arnkoff D. B. Glass C. R. (2011). Conceptualizing mindfulness and acceptance as components of psychological resilience to trauma. Trauma Violence Abuse 12, 220–235. doi: 10.1177/1524838011416375, PMID: 21908440

[ref70] Thrash T. M. Elliot A. J. Maruskin L. A. Cassidy S. E. (2010). Inspiration and the promotion of well-being: tests of causality and mediation. J. Pers. Soc. Psychol. 98, 488–506. doi: 10.1037/a0017906, PMID: 20175626

[ref9001] Tran U. S. Birnbaum L. Burzler M. A. Hegewisch U. J. C. Ramazanova D. Voracek M. (2022). Self-reported mindfulness accounts for the effects of mindfulness interventions and nonmindfulness controls on self-reported mental health: A preregistered systematic review and three-level meta-analysis of 146 randomized controlled trials. Psychol. Bull. 148, 86–106. doi: 10.1037/bul0000359, PMID: 32210868

[ref71] Wang Y. B. Tian T. Wang J. J. (2022). A mediating model of mindfulness, sense of purpose in life and mental health among Chinese graduate students. BMC psychol. 10:90. doi: 10.1186/s40359-022-00799-4, PMID: 35387684 PMC8985752

[ref72] Watson D. Clark L. A. Tellegen A. (1988). Development and validation of brief measures of positive and negative affect: the PANAS scales. J. Pers. Soc. Psychol. 54, 1063–1070. doi: 10.1037/0022-3514.54.6.1063, PMID: 3397865

[ref73] Wen Z. L. Huang B. B. Tang D. D. (2018). Preliminary work for modeling questionnaire data. J. Psychol. Sci. 41, 204–210. doi: 10.16719/j.cnki.1671-6981.20180130

[ref74] Westerhof G. J. Keyes C. L. M. (2010). Mental illness and mental health: the two continua model across the lifespan. J. Adult Dev. 17, 110–119. doi: 10.1007/s10804-009-9082-y, PMID: 20502508 PMC2866965

[ref75] Wu Z. Y. (2016). Development of decision-making model of exercise adherence: the added value of self-regulatory processes and affective experience. Beijing: Beijing Sport University.

[ref76] Wu D. Yu L. W. Yang T. Z. Cottrell R. D. Peng S. H. Guo W.. (2020). The impacts of uncertainty stress on mental disorders of Chinese college students: evidence from a nationwide study. Front. Psychol. 11:243. doi: 10.3389/fpsyg.2020.00243, PMID: 32210868 PMC7075936

[ref77] Xu W. (2020). Trait mindfulness: a new visual angle for the study of exercise psychology. J. Chengdu Sport Univ. 46, 94–99. doi: 10.15942/j.jcsu.2020.01.015

[ref78] Xu W. (2021). Physical exercise and depression among college students: the multiple mediating roles of mindfulness and social support. Soc. Sci. 2, 148–155.

[ref79] Ye W. F. Lu J. M. Liu X. S. Wu Y. X. (2019). The relationship between goal contents and subjective well-being among Chinese college students:a moderated mediation model. Chinese. J. Psychol. Sci. 42, 379–386. doi: 10.16719/j.cnki.1671-6981.20190218

[ref80] Zenner C. Herrnleben-Kurz S. Walach H. (2014). Mindfulness-based interventions in schools—a systematic review and meta-analysis. Front. Psychol. 5:603. doi: 10.3389/fpsyg.2014.00603, PMID: 25071620 PMC4075476

[ref81] Zhang Y. Cui L. J. Li K. Q. Jiang Q. P. Sun X. L. Gao L. H.. (2008). Supplemented edition of the general health questionnaire (GHQ-12) in epidemiological survey of mental illness. Chin. Ment. Health J. 3, 189–192.

[ref82] Zhang Q. J. Lu J. L. Quan P. (2021). Application of the dual-factor model of mental health among Chinese new generation of migrant workers. BMC psychol. 9:188. doi: 10.1186/s40359-021-00693-5, PMID: 34847959 PMC8630867

[ref83] Zhou H. Long L. R. (2004). Statistical remedies for common method biases. Adv. Psychol. Sci. 6, 942–950.

[ref84] Zhu Y. Zhao Y. Zhou Y. Wu J. (2019). Resilience in organizations: construction of protective resources from psychological and systematic perspective. Adv. Psychol. Sci. 27, 357–369. doi: 10.3724/SP.J.1042.2019.00357

